# Indicators of abdominal size relative to height associated with sex, age, socioeconomic position and ancestry among US adults

**DOI:** 10.1371/journal.pone.0172245

**Published:** 2017-03-01

**Authors:** Henry S. Kahn, Kai McKeever Bullard

**Affiliations:** Division of Diabetes Translation, Centers for Disease Control and Prevention, Atlanta, Georgia, United States of America; Medical University of Vienna, AUSTRIA

## Abstract

**Background/Objectives:**

The supine sagittal abdominal diameter (SAD) and standing waist circumference (WC) describe abdominal size. The SAD/height ratio (SADHtR) or WC/height ratio (WHtR) may better identify cardiometabolic disorders than BMI (weight/height^2^), but population-based distributions of SADHtR and WHtR are not widely available. Abdominal adiposity may differ by sociodemographic characteristics.

**Subjects/Methods:**

Anthropometry, including SAD by sliding-beam caliper, was performed on 9894 non-pregnant adults ≥20 years in the US National Health and Nutrition Examination Surveys of 2011–2014. Applying survey design factors and sampling weights, we estimated nationally representative SADHtR and WHtR distributions by sex, age, educational attainment, and four ancestral groups.

**Results:**

The median (10th percentile, 90th percentile) for men’s SADHtR was 0.130 (0.103, 0.165) and WHtR 0.569 (0.467, 0.690). For women, median SADHtR was 0.132 (0.102, 0.175) and WHtR 0.586 (0.473, 0.738). Medians for SADHtR and WHtR increased steadily through age 79. The median BMI, however, reached maximum values at ages 40–49 (men) or 60–69 (women) and then declined. Low educational attainment, adjusted for age and ancestry, was associated with elevated SADHtR more strongly than elevated BMI. While non-Hispanic Asians had substantially lower BMI compared to all other ancestral groups (adjusted for sex, age and education), their relative reductions in SADHtR and WHtR, were less marked.

**Conclusions:**

These cross-sectional data are consistent with monotonically increasing abdominal adipose tissue through the years of adulthood but decreasing mass in non-abdominal regions beyond middle age. They suggest also that visceral adipose tissue, estimated by SADHtR, expands differentially in association with low socioeconomic position. Insofar as Asians have lower BMIs than other populations, employing abdominal indicators may attenuate the adiposity differences reported between ancestral groups. Documenting the distribution and sociodemographic features of SADHtR and WHtR supports the clinical and epidemiologic adoption of these adiposity indicators.

## Introduction

Clinical assessments of adiposity commonly depend on the body mass index (BMI, weight/height^2^) as a convenient indicator of fatness relative to height [1]. While BMI is more useful than body weight alone, its dependence on non-specified “mass” may obscure differing contributions made by fat and lean tissues [2] or fail to recognize the health consequences attributable to variations in body fat distribution [3, 4]. Despite these limitations for clinical screening and physiologic research, BMI has served those who monitor population-based, categorical “overweight” and “obesity” in adults across time and different cultures [5, 6]. For over three decades, population reference data on BMI in the United States have been published periodically from the National Health and Nutrition Examination Survey (NHANES). Most recently, NHANES has reported the detailed BMI distribution for adult women and men during survey years 2011–2014 [7].

Reports based on the BMI describe an increasing prevalence of obesity worldwide [8]. Serial surveys obtained across at least two decades have documented disproportionate increases in the standing waist circumference (WC) for a given level of BMI [9–11]. Since WC is correlated with abdominal adipose tissue, total body fat, and with various markers of cardiometabolic risk [12], any increases in WC that are disproportionate (i.e., beyond increases in total body mass) may carry adverse health consequences for the population. In order to simplify the concept of abdominal adiposity, WC was transformed into the WC/height ratio (WHtR) more than two decades ago [13, 14] so as to acknowledge the variation in adult stature [15].

The supine sagittal abdominal diameter (SAD, “abdominal height”), also correlated with abdominal adipose tissue, has been proposed as an alternative body dimension to the WC. The SAD, which can be measured with a sliding-beam caliper, may estimate the expansion of the visceral (intra-abdominal) adipose compartment more specifically than the WC [16–20]. SAD has been transformed similarly into the SAD/height ratio (SADHtR) [21, 22].

Early studies reported that inter- and intra-observer variations in measurement of WC or SAD were comparable and small in comparison to the variation between patients [23, 24]. A more recent report suggested that SAD (measured supine with flexed hips) had higher reliability than supine WC especially among those with BMI ≥26 kg/m^2^ [25]. However, each of these 3 studies was limited by its small study sample.

The clinical use of SADHtR [26, 27] or WHtR [26–30] can improve recognition of cardiometabolic risk when compared to the use of BMI. Despite growing interest in low-cost assessments of abdominal adiposity, current population distributions of SADHtR and WHtR are not widely available. This paper summarizes community-based estimates (reference data) of how these two adiposity indicators are currently distributed among adults (ages 20+ years) in the United States by sex and decades of age. We also point out variations in abdominal adiposity associated with low educational attainment, low family income, and four major ancestral groups. Our report concludes with commentary that acknowledges the few, community-based distributions of adult SADHtR and WHtR that were published in earlier years and from other countries.

## Participants and methods

Anthropometric data were obtained from the NHANES, an ongoing, nationally representative, cross-sectional survey of the resident civilian, non-institutionalized, US population [31]. Participants chosen for NHANES undergo home interviews followed by standardized anthropometric and laboratory assessments in mobile examination centers. The NHANES protocol was approved by the Research Ethics Review Board of the National Center for Health Statistics; participants provided informed consent.

Our report covers non-pregnant adults (age ≥20 years) examined in 2011–2014. Weight, height and WC (standing position, by tape measure just above the uppermost lateral border of the ilium) were obtained by established methods [32]. WHtR was calculated as WC [cm]/height [cm]. BMI was calculated as weight [kg]/height [m]^2^. During 2011–2014 NHANES did not measure circumferences of the hip or midthigh.

The SAD measurement used a portable, sliding-beam caliper (Holtain, Ltd, Wales, UK) [32]. Supine participants rested on a lightly padded exam table with their hips in flexed position as the examiner marked the level of their iliac crests. The lower arm of the caliper was then positioned under the small of the back, and the upper arm was raised above the belly in alignment with their iliac-crest level. The examiner asked the participant to inhale gently, slowly let the air out, and then relax. The examiner then lowered the caliper’s upper arm, letting it lightly touch the abdomen but without compressing it. The SAD value was read directly from a centimeter scale on the caliper shaft, recorded in duplicate to the nearest 0.1 cm. For 94.2% of adults we defined SAD as the mean of two initial measurements; for 5.8% (with initial SAD discrepancy >0.5 cm) we used the mean of up to 4 measurements [33]. SADHtR was calculated as SAD [cm]/height [cm].

Of 10,785 non-pregnant adults attending the examination centers, 10,636 provided BMI values, 9,907 provided SADHtR, and 10,116 provided WHtR. We restricted our primary descriptive sample to include only the 9,894 adults who had values for both SADHtR and WHtR (91.7% of examination attendees). These examinees represented approximately 208.5 million US adults. Multivariable regression models were restricted to 9,882 of these who had values also for BMI, as well as SADHtR and WHtR (91.6% of attendees).

### Sociodemographic covariates

As derived from self-reported race and Hispanic origin (ethnicity) we identified five, mutually exclusive, ancestral groups as non-Hispanic white, non-Hispanic black, Hispanic, non-Hispanic Asian, and “others” [34]. For comparison with persons who attended some college or higher education, we categorized low educational attainment as either less than high school completion or high school graduation (may include passing the General Educational Development tests). For comparison with persons having a family income of at least 200% of poverty guidelines, we categorized low income as either <100% of poverty guidelines or 100% to <200% of poverty guidelines; an additional category of “unknown” income was included in these models [35].

### Statistical methods

NHANES selected participants through a complex, multistage-probability design requiring a sampling weight for each participant. We used SAS (release 9.3; SAS Institute Inc.) and SUDAAN (PROC DESCRIPT and PROC REGRESS, release 11.1; RTI International) to account for the complex design and sampling weights so that characteristics of the represented population could be correctly described.

To describe the associations of socioeconomic variables with adiposity we created sex-specific, multivariable, linear regression models having outcomes of SADHtR, WHtR or BMI. All multivariate models were age-adjusted in 7 categories (decades) of which the highest was 80+ years. As generated by these linear regression models, a beta coefficient (sometimes called *regression coefficient*) estimates the amount by which the outcome differs for persons in a designated socioeconomic category (e.g., “less than high-school completion” or “high school graduation”) compared to persons in the reference category (e.g., “persons who attended some college or higher education”). In these separate models we employed the adjusted Wald F statistic to estimate the confidence assigned to the contrasts (summary effect) within the 3-level variables for education or for family income regarding their influence on the observed value for the adiposity indicator. The adjusted Wald F is based on the Wald chi-squared with adjusted denominator degrees of freedom. A higher adjusted Wald F value means a higher likelihood of the variable showing statistical significance.

To describe how each of four major ancestral groups differed from the remaining US population, we built a series of similar linear regression models having outcomes of SADHtR, WHtR or BMI. As generated by these linear regression models, a beta coefficient estimates the amount by which the outcome differs for persons in a designated ancestral group (e.g., “Non-Hispanic whites”) compared to persons in the reference category (e.g., “sum of all the ancestries excluding Non-Hispanic whites”). As used here, the adjusted Wald F statistic estimates the confidence assigned to each 2-level variable of ancestral contrasts.

## Results

For SADHtR, among US men (sample N = 4,949) the population mean was 0.1326 (SE 0.0007) and median was 0.130 (10^th^ percentile = 0.103; 90^th^ percentile = 0.165). Among women (sample N = 4,945) the SADHtR population mean was 0.1357 (SE 0.0007) and median was 0.132 (10^th^ percentile 0.102; 90^th^ percentile = 0.175). Greater detail for SADHtR percentiles 5 through 95 is available from Tables A and B in S1 File.

For WHtR, among US men the population mean was 0.5770 (SE 0.0023) and median was 0.569 (10^th^ percentile = 0.467; 90^th^ percentile = 0.690). Among women the WHtR population mean was 0.5972 (SE 0.0024) and median was 0.586 (10^th^ percentile 0.473; 90^th^ percentile = 0.738). Greater detail for WHtR percentiles is available from Tables C and D in S1 File.

### Age and sex

**Fig 1:**
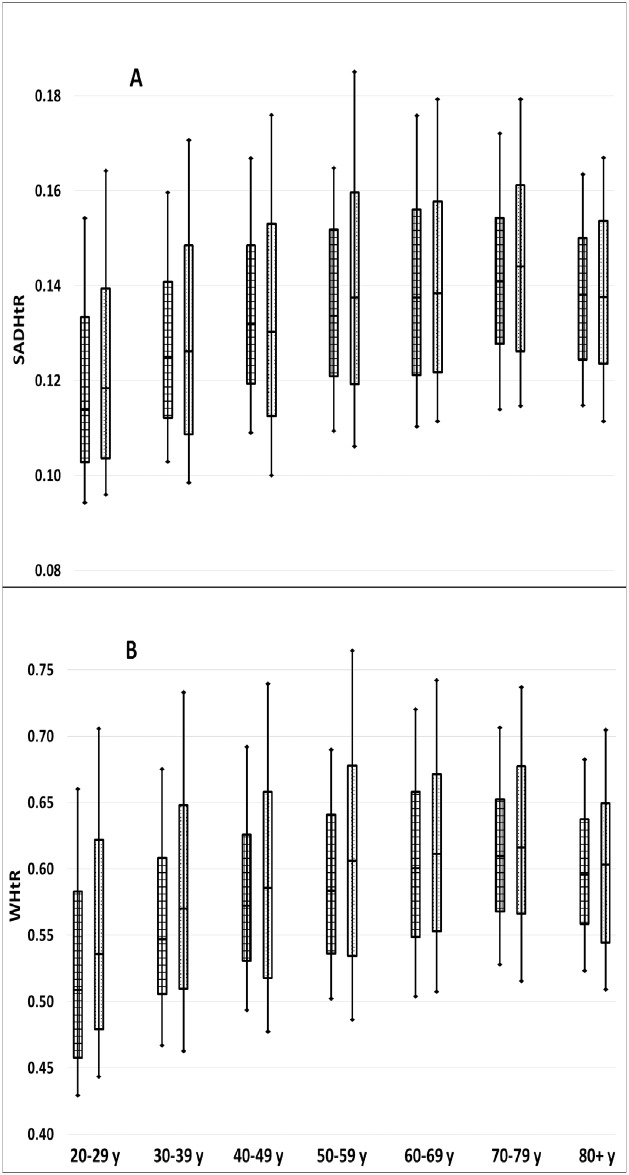
Distributions of [A] SAD/height ratio (SADHtR) and [B] waist circumference/height ratio (WHtR) by age group and sex (men on left, women on right). Boxes indicate population percentiles 25, 50, 75; whiskers identify p10 and p90.

**Fig 2:**
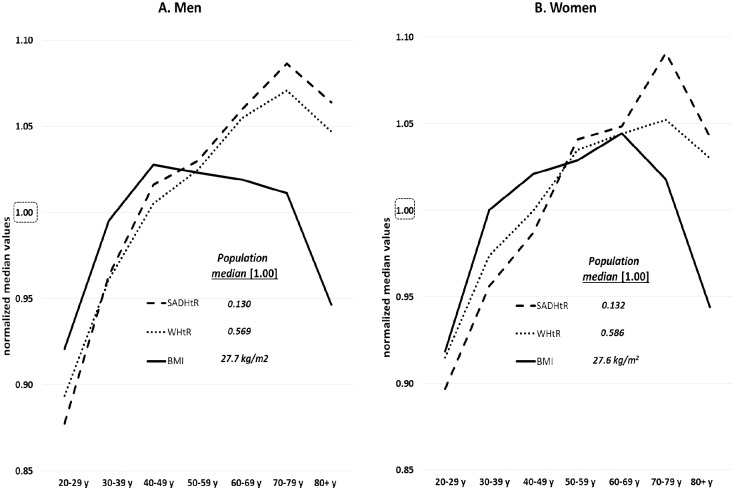
Age-specific medians plotted by decade for SADHtR, WHtR, and BMI. Median values were normalized with reference to the sex-specific median [1.00] estimated for the full population of [A] men or [B] women at ages 20+ years.

Fig 1 demonstrates that for both abdominal adiposity indicators the women had a wider distribution of values than the men across the complete adult age range. The sex difference (men<women) in median values was less prominent for SADHtR than for WHtR. When median values of SADHtR and WHtR in each age group were normalized with reference to the sex-specific median of men or women at all ages (20+ y), these two indicators demonstrated a roughly parallel increase with age from 20–29 through 70–79 y (Fig 2). The relative increment, however, across these six age decades was slightly steeper for SADHtR than WHtR. Age-related variation in BMI showed a different pattern. After rises in early adulthood (similar to relative rises in abdominal indicators), the men’s BMI was lower after age 50, then more so after about 79 y. For women’s BMI there was a marked increase preceding age 30–39 (steeper than the relative rises in abdominal indicators), a more gradual increase up to age 60–69, followed thereafter by steep declines (Fig 2).

### Socioeconomic position

**Fig 3:**
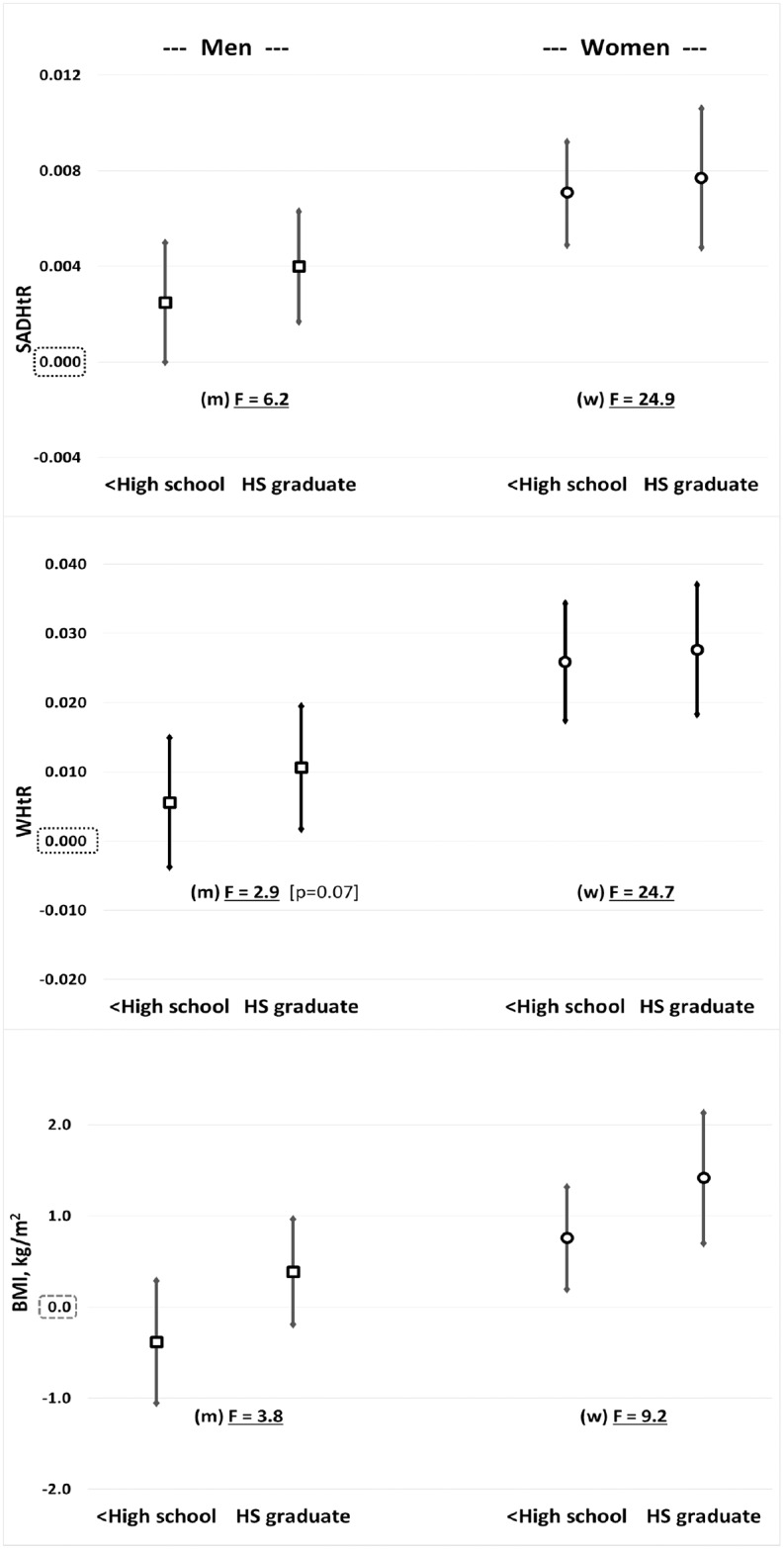
Sex-specific beta coefficients (95% confidence range) associated with low educational attainment (less than high-school completion or high school graduation) compared with persons who attended some college or higher education. Separate models, each adjusted for age and ancestral groups, were prepared for [top] SADHtR, [middle] WHtR, and [bottom] BMI. F indicates the adjusted Wald F statistic, a summary effect describing confidence that contrasts in educational attainment (3-levels) have influenced the observed value for the adiposity indicator.

Compared to those who attained education beyond high school, after adjustments for age and ancestry, men with less than high school completion had elevated SADHtR, but no significant difference in WHtR or BMI (Fig 3). Men who attained only high school graduation had greater SADHtR and WHtR, but not greater BMI. The summary effect on adiposity of men’s low educational attainment was greater when estimated by SADHtR (adjusted Wald F = 6.2, p = 0.006) than by the alternative indicators. For women, low education was associated with elevation of all three adiposity indicators, but the summary effect was greatest when estimated by SADHtR (adjusted Wald F = 24.9, p<0.0001) or by WHtR (adjusted Wald F = 24.7, p<0.0001).

In a set of similar statistical models we compared adiposity among persons with low family income (<100% of poverty guidelines or 100% to <200% of poverty guidelines) to those with family income ≥200% of poverty guidelines. These models included an additional category of “unknown” family income (7% of participants). Among men in the lowest income group, BMI was significantly *lower* than the reference income category, but for this most impoverished group there were no significant differences for SADHtR or WHtR (S1 Fig). Men at 100% to <200% of poverty guidelines had elevated SADHtR and WHtR, but no difference in their BMI. Among women, both categories of low family income were associated with elevations of all adiposity indicators, with the greatest summary effects seen for SADHtR (adjusted Wald F = 27.6, p<0.0001) and WHtR (adjusted Wald F = 26.8, p<0.0001).

### Ancestral groups

**Fig 4:**
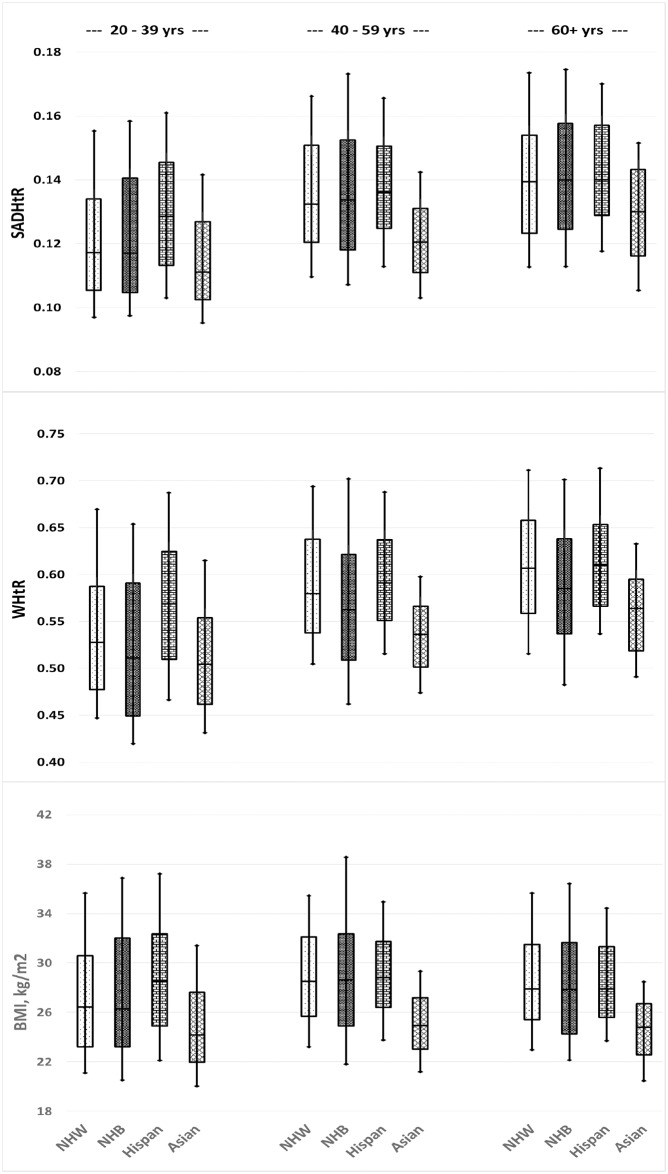
Men’s distributions of SAD/height ratio (SADHtR), waist circumference/height ratio (WHtR), and body mass index by 4 ancestral groups and 3 age groups. Boxes indicate population percentiles 25, 50, 75; whiskers identify p10 and p90. NHW = Non-Hispanic whites; NHB = Non-Hispanic blacks; Hispan = Hispanics; Asian = Non-Hispanic Asians.

**Fig 5:**
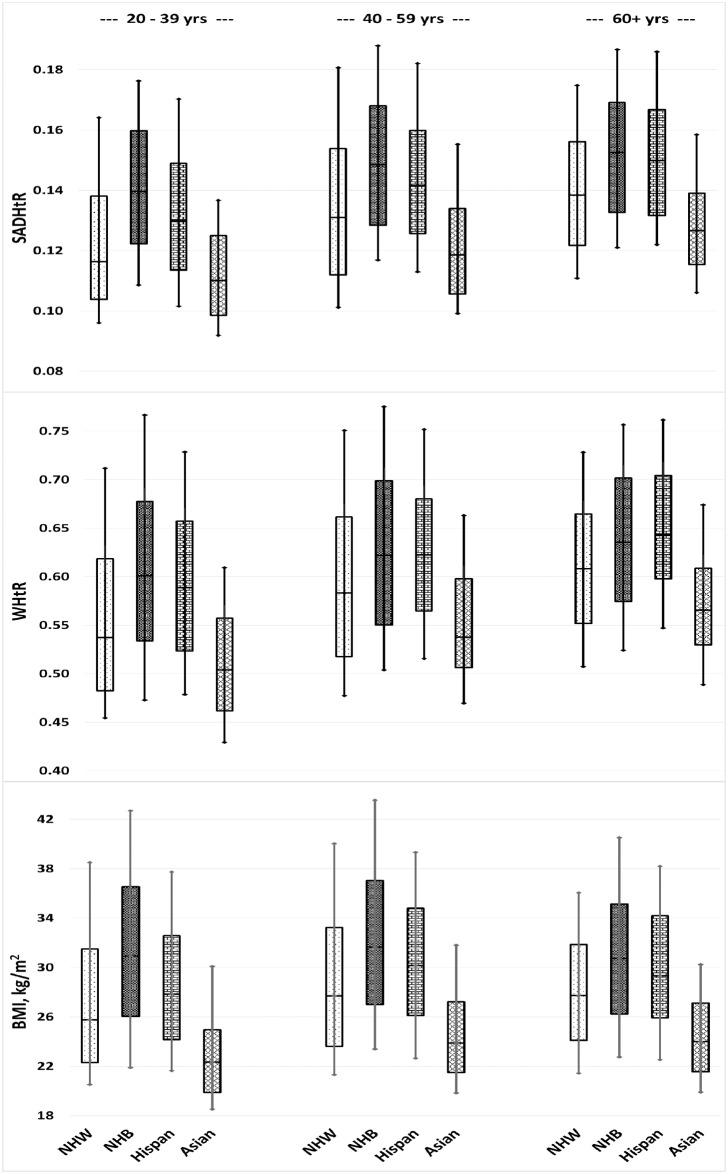
Women’s distributions of SAD/height ratio (SADHtR), waist circumference/height ratio (WHtR), and body mass index by 4 ancestral groups and 3 age groups. Boxes indicate population percentiles 25, 50, 75; whiskers identify p10 and p90. NHW = Non-Hispanic whites; NHB = Non-Hispanic blacks; Hispan = Hispanics; Asian = Non-Hispanic Asians.

Within our represented US adult population the identified ancestral proportions were non-Hispanic white (66.4%), non-Hispanic black (11.1%), Hispanic (14.6%), non-Hispanic Asian (5.2%), and others (2.7%). Across three age strata, we have displayed the distributions of SADHtR, WHtR, and BMI found in the four largest ancestral groups for men (Fig 4) and women (Fig 5).

Non-Hispanic white men, compared to men remaining in the total of all other groups, had no significant differences in mean SADHtR, WHtR, or BMI (Table 1). For non-Hispanic white women, however, the means of all three of the adiposity indicators were modestly lower than those found for the total of all other women; this relative reduction was greatest for SADHtR (adjusted Wald F = 31.5; Table 1).

**Table 1:** Adiposity differences among US adults (according to SADHtR, WHtR, or BMI) for four ancestral groups, each compared to all other ancestries.

		Beta coefficient	SE beta	Adjusted Wald F (ancestral contrast)
**Non-Hispanic Whites (*vs* all others)**				
***Men --***	**SADHtR**	-0.0011	0.0008	1.8
	**WHtR**	0.004	0.004	1.4
	**BMI, *kg/m***^***2***^	0.08	0.24	0.1
***Women --***	**SADHtR**	-0.0062	0.0011	31.5***
	**WHtR**	-0.015	0.004	15.0**
	**BMI, *kg/m***^***2***^	-0.91	0.28	10.4*
**Non-Hispanic Blacks (*vs* all others)**				
***Men --***	**SADHtR**	0.0006	0.0009	0.4
	**WHtR**	-0.020	0.004	32.1***
	**BMI, *kg/m***^***2***^	0.14	0.26	0.3
***Women --***	**SADHtR**	0.0138	0.0009	212.2***
	**WHtR**	0.033	0.004	90.4***
	**BMI, *kg/m***^***2***^	3.43	0.28	150.9***
**Hispanics (*vs* all others)**				
***Men --***	**SADHtR**	0.0049	0.0013	14.9**
	**WHtR**	0.023	0.005	19.6**
	**BMI, *kg/m***^***2***^	1.00	0.35	7.9*
***Women --***	**SADHtR**	0.0053	0.0015	12.6*
	**WHtR**	0.022	0.005	16.3**
	**BMI, *kg/m***^***2***^	0.87	0.35	6.1
**Non-Hispanic Asians (*vs* all others)**				
***Men --***	**SADHtR**	-0.0113	0.0009	147.2***
	**WHtR**	-0.044	0.003	207.8***
	**BMI, *kg/m***^***2***^	-3.66	0.21	317.9***
***Women --***	**SADHtR**	-0.0145	0.0012	153.5***
	**WHtR**	-0.053	0.004	218.4***
	**BMI, *kg/m***^***2***^	-4.96	0.27	341.6***

* p <0.01;

** p < 0.001;

*** p <0.0001

Models are adjusted for age (7 categories) and education (3 categories).

For non-Hispanic black men (*vs* all others) we found a reduced mean for WHtR, but no difference for SADHtR or BMI. For non-Hispanic black women, the means of all three indicators were elevated compare to those for all other women, with the greatest increment observed for SADHtR (adjusted Wald F = 212.2; Table 1). As estimated by their interquartile ranges or by their ranges between percentiles 10 and 90, we noted that the variation in BMI was wider for non-Hispanic black men (Fig 4) and women (Fig 5) than the variation seen within each of the other three major ancestral groups. The ranges in WHtR and SADHtR among non-Hispanic blacks (*vs* all others) also showed wider variation, but less markedly than the variations in BMI.

Hispanic men and women (*vs* all others) had elevated mean values of all adiposity indicators, with a more pronounced increment for the abdominal indicators than for the BMI (Table 1).

Non-Hispanic Asian men and women (*vs* all others) had lower mean values of all adiposity indicators, with BMI showing the most prominent decrement. In models that included both sexes (with sex adjustment), the non-Hispanic Asians had lower mean BMI compared to the total of all other ancestries (adjusted Wald F = 520.0), along with less marked decrements in SADHtR (adjusted Wald F = 233.8) and WHtR (adjusted Wald F = 357.4).

## Discussion

Anthropometric reference data for adults are empirical, normative values associated with a given place and time. Regarding the SADHtR, a population distribution was first reported nationally from a survey in 2000–2001 of older Finnish adults (ages 30+ years) who were almost entirely of white, northern European ancestry [36]. We are unaware of any other population-based descriptions of SADHtR. From both Finland and the US these cross-sectional data, stratified by sex, demonstrate the same pattern of continuous increases with ageing through at least 69 y. In other adult samples of diverse ancestries, abdominal imaging studies have shown likewise that visceral adipose tissue increases with age more than the subcutaneous adipose depot [37, 38]. Taken together, these observations describe a generalized, monotonic increase of abdominal adipose tissue, especially in the visceral depot, through nearly all the years of adulthood. The relative declines in BMI beyond middle age (Fig 3), however, indicate a likelihood among the elderly of decreasing mass in non-abdominal regions.

Compared to the earlier Finnish report of SADHtR, the more recent US data reveal larger SADHtR values obtained for the same age strata. Within each sex, the US SADHtR values approximated the Finnish SADHtR values found for the next oldest decade of age. These contrasts could be explained by the rising adiposity trend worldwide during recent years or by inherent cultural and environmental differences between Finland and the US.

Regarding the WHtR, adult population statistics were published for the United States in 1999–2004 (ages 20+; N = 12,936). In that earlier time period the WHtR for adult US men was 0.565 and women was 0.575, but these summary values did not show stratifications by age [39]. Subsequent adult population statistics on WHtR have been reported from China (in 2005–2007, ages 35–70; N = 43,841) [40], Canada (in 2007–2009, ages 20–69; N = 10,605) [9], and Colombia (in 2010, ages 20–64; N = 83,220) [41]. Of these large reported surveys, only the Colombian publication included breakdowns by age group. A comparison of these various reports is complicated by variation in anthropometric method or sample frames that differ by age or urban-rural status [42]. However, the limited data are consistent with previous observations that the WC, as well as the WHtR, has increased more among women than men during recent decades [9–11].

We are unaware of any previous report relating SADHtR to a marker of socioeconomic position, but adult WHtR has been associated inversely with educational attainment in one prior study [43]. SAD alone (without division by height), however, was inversely associated with educational attainment among Finnish adults, more strongly for women than men [36]. Similarly, adult WC alone was inversely associated with education, especially for women, in large studies from four other countries [44–47]. Our report confirms that a similar inverse association with education is found when SADHtR or WHtR is the adiposity indicator (Fig 3). We demonstrate further that either abdominal adiposity indicator had a stronger inverse association than BMI with educational attainment.

Associations between low income and adiposity are infrequently reported, perhaps because of difficulties in ascertaining the income level of individual participants. In this regard the NHANES calculation of poverty-income ratio—despite the absence of income information in about 7% of participants—demonstrates that women’s poverty had strongly inverse associations with SADHtR and WHtR. For men, however, we found that the lowest income level was associated with no change in abdominal adiposity but with a decrement of about 1 BMI unit (S1 Fig). Why the men’s pattern differs so greatly from the women’s merits further study.

A unique contribution of our report is its demonstration of substantial differences between ancestral groups in the empirical distributions of SADHtR and WHtR (Figs 4 [men] and 5 [women]). Among these box-and-whisker plots we present also an updated description of ancestral differences in the BMI distribution. A Scientific Statement from the American Heart Association recently addressed these distinctions with regard to BMI [48]. Given that some ancestral groups have BMI distributions substantially larger or smaller than the majority non-Hispanic white population, the authors questioned the value of applying universal BMI threshold values, such as 25 or 30 kg/m^2^, to the assessment of diverse adult populations. Our models adjusted for age and education confirm that the ancestral differences in BMI are robust, especially as they apply to the lower BMI values that distinguish the non-Hispanic Asian population from all others (Table 1). The Asian differences are also substantial with regard to WHtR and SADHtR, although the distinctions appear to be attenuated (reduced Wald F statistic for ancestral contrast) when compared to the Asian differences in BMI. Some authorities have recommended that for clinical screening purposes a WHtR boundary should be set simply at 0.5, and that this threshold would be suitable for all ethnic groups [49]. We found among US adults, however, that the application of this universal screening threshold for WHtR would designate more than half of non-Hispanic Asians, and larger proportions of the remaining population, as being at increased health risk (Figs 4 and 5). Thus, the proposed WHtR threshold of 0.5 may be impractical for application in the contemporary United States [28]. To our knowledge, no universal threshold values for SADHtR have been proposed. Any anthropometric thresholds recommended for specific ancestral groups might still misclassify risk status because of confounding by sex [50], socioeconomic position, and the ambiguities inherent in terms such as “Asian”, “Hispanic”, “black”, and “white” [48].

A limitation of our report is related to the variety of available protocols for measurement of WC. The NHANES protocol, adopted also by the US National Institutes of Health and some Canadian organizations, specifies that the tape measure should be applied just above the uppermost lateral border of the ilium [32]. An alternative WC protocol in wide use, recommended by the World Health Organization (WHO), specifies that the tape measure should be applied midway between the inferior margin of the last rib and the crest of the ilium [6]. The reproducibility of the adult WC measured by either of these protocols is high, but the resulting WC values may differ slightly, most notably for women [51]. These alternative definitions of the WC result in small differences of the WHtR values that may influence the prevalence estimates of “high WHtR” or the WHtR percentile positions. Among our cited distributions of adult WHtR obtained from outside the US, the recent Chinese article [40] depended on WC measured by the NHANES protocol while the Canadian [9] and Colombian [41] articles used the WHO protocol.

The anthropometric protocol for SAD measurement in Finland’s survey was defined by the iliac crests [36], the same as our US protocol in NHANES. Evaluations of four different SAD protocols concluded that this site for measuring SAD was the best correlated with markers of cardiometabolic risk [52, 53].

We understand there are many reasons why BMI will continue to be calculated and reported as a primary index of weight status and health risk. In settings where a high quality scale is available, the simplicity of standardized weight measurement has its appeal. Whether patients and research participants will reliably disrobe and put down their various heavy objects is another question. It is likely, however, that some clinicians, researchers, and epidemiologists will come to prefer the SADHtR and WHtR as inexpensive markers more specifically focused on abdominal adiposity. Agreement on a standardized, common protocol for WC measurement will encourage practitioners to familiarize themselves with meaningful use of a tape measure and calculation of the WHtR. The NHANES success with standardized caliper measurements of SAD should build confidence that the SAD can also be obtained easily and interpreted by calculation of the SADHtR. The documentation of how WHtR and SADHtR have recently been distributed among US adults may help to promote the adoption of these abdominal adiposity indicators.

## Supporting information

S1 File Adult distributions (SADHtR, WHtR) by sex. Table A. SAD/height ratio (SADHtR) for men aged 20+
Table B. SAD/height ratio (SADHtR) for women aged 20+
Table C. Waist circumference/height ratio (WHtR) for men aged 20+
Table D. Waist circumference/height ratio (WHtR) for women aged 20+.
(DOCX)

S1 Fig Sex-specific beta coefficients (95% confidence range) associated with low family income (<100% of poverty guidelines or 100% to <200% of poverty guidelines) compared to persons with family income ≥200% of poverty guidelines. Separate models, each adjusted for age and ancestral groups, were prepared for [top] SADHtR, [middle] WHtR, and [bottom] BMI. F indicates the adjusted Wald F statistic, a summary effect describing confidence that contrasts in family income (3-levels) have influenced the observed value for the adiposity indicator. These models include a category of persons (7%) with unknown family income.
(TIF)
