# Supplementary material for: Indicators of abdominal size relative to height associated with sex, age, socioeconomic position and ancestry among US adults
Source: PLoS One. 2017 Mar 1;12(3):e0172245. doi: 10.1371/journal.pone.0172245 (PMC5332027; doi:10.1371/journal.pone.0172245)
Supplement: S1 File — Table A. SAD/height ratio (SADHtR) for men aged 20+ Table B. SAD/height ratio (SADHtR) for women aged 20+ Table C. Waist circumference/height ratio (WHtR) for men aged 20+ Table D. Waist circumference/height ratio (WHtR) for women aged 20+. (DOCX) [file pone.0172245.s001.docx]

**S1 Supporting tables. Adult distributions (SADHtR, WHtR) by sex.**

**Table A**. **SAD/height ratio (SADHtR) for men aged 20 and over and number of examined persons, mean, standard error of the mean, and selected percentiles by ancestry and age: United States, 2011-2014. Estimated population = 103.4 million men.**

|  |  |  |  |  |  | **Percentile in population** | | | | | | |
| --- | --- | --- | --- | --- | --- | --- | --- | --- | --- | --- | --- | --- |
| **Race and ancestry and age** | | | **Number of examined persons** | **Population mean** | **Standard error of the mean** | **5th** | **10th** | **25th** | **Median 50th** | **75th** | **90th** | **95th** |
| ***All ancestries**** | |  |  |  |  |  |  |  |  |  |  |  |
| **20 + years [total]** | |  | 4949 | 0.1326 | 0.0007 | 0.098 | 0.103 | 0.115 | 0.130 | 0.148 | 0.165 | 0.178 |
| **20-29 years** | |  | 911 | 0.1201 | 0.0015 | 0.091 | 0.094 | 0.103 | 0.114 | 0.133 | 0.154 | 0.167 |
| **30-39 years** | |  | 886 | 0.1286 | 0.0009 | 0.100 | 0.103 | 0.112 | 0.125 | 0.141 | 0.160 | 0.170 |
| **40-49 years** | |  | 835 | 0.1352 | 0.0009 | 0.102 | 0.109 | 0.119 | 0.132 | 0.149 | 0.167 | 0.178 |
| **50-59 years** | |  | 819 | 0.1371 | 0.0010 | 0.103 | 0.109 | 0.121 | 0.134 | 0.152 | 0.165 | 0.179 |
| **60-69 years** | |  | 827 | 0.1405 | 0.0017 | 0.104 | 0.110 | 0.121 | 0.138 | 0.156 | 0.176 | 0.189 |
| **70-79 years** | |  | 440 | 0.1421 | 0.0013 | 0.107 | 0.114 | 0.128 | 0.141 | 0.154 | 0.172 | 0.182 |
| **80 + years** | |  | 231 | 0.1384 | 0.0013 | 0.109 | 0.115 | 0.124 | 0.138 | 0.150 | 0.164 | 0.172 |
| ***Non-Hispanic white*** | |  |  |  |  |  |  |  |  |  |  |  |
| **20 + years [total]** | |  | 1975 | 0.1328 | 0.0008 | 0.098 | 0.103 | 0.115 | 0.129 | 0.149 | 0.165 | 0.179 |
| **20-39 years** | |  | 700 | 0.1222 | 0.0015 | 0.092 | 0.097 | 0.105 | 0.117 | 0.134 | 0.155 | 0.167 |
| **40-59 years** | |  | 631 | 0.1363 | 0.0010 | 0.103 | 0.110 | 0.120 | 0.132 | 0.151 | 0.166 | 0.179 |
| **60 + years** | |  | 644 | 0.1408 | 0.0013 | 0.105 | 0.113 | 0.123 | 0.139 | 0.154 | 0.174 | 0.185 |
| ***Non-Hispanic black*** | |  |  |  |  |  |  |  |  |  |  |  |
| **20 + years [total]** | |  | 1145 | 0.1329 | 0.0008 | 0.097 | 0.102 | 0.113 | 0.129 | 0.149 | 0.169 | 0.184 |
| **20-39 years** | |  | 388 | 0.1242 | 0.0011 | 0.093 | 0.098 | 0.105 | 0.117 | 0.141 | 0.158 | 0.170 |
| **40-59 years** | |  | 373 | 0.1379 | 0.0014 | 0.101 | 0.107 | 0.118 | 0.134 | 0.153 | 0.173 | 0.189 |
| **60 + years** | |  | 384 | 0.1421 | 0.0016 | 0.104 | 0.113 | 0.125 | 0.140 | 0.158 | 0.175 | 0.188 |
| ***Hispanic*** |  |  |  |  |  |  |  |  |  |  |  |  |
| **20 + years [total]** | |  | 1041 | 0.1350 | 0.0011 | 0.101 | 0.107 | 0.119 | 0.134 | 0.148 | 0.165 | 0.176 |
| **20-39 years** | |  | 376 | 0.1308 | 0.0016 | 0.097 | 0.103 | 0.113 | 0.129 | 0.146 | 0.161 | 0.171 |
| **40-59 years** | |  | 369 | 0.1385 | 0.0011 | 0.105 | 0.113 | 0.125 | 0.136 | 0.151 | 0.166 | 0.176 |
| **60 + years** | |  | 296 | 0.1434 | 0.0014 | 0.114 | 0.118 | 0.129 | 0.140 | 0.157 | 0.170 | 0.181 |
| ***Non-Hispanic Asian*** | |  |  |  |  |  |  |  |  |  |  |  |
| **20 + years [total]** | |  | 634 | 0.1206 | 0.001 | 0.094 | 0.098 | 0.107 | 0.119 | 0.133 | 0.145 | 0.152 |
| **20-39 years** | |  | 249 | 0.1158 | 0.0014 | 0.090 | 0.095 | 0.102 | 0.111 | 0.127 | 0.142 | 0.151 |
| **40-59 years** | |  | 236 | 0.1216 | 0.001 | 0.097 | 0.103 | 0.111 | 0.121 | 0.131 | 0.142 | 0.149 |
| **60 + years** | |  | 149 | 0.1297 | 0.0014 | 0.098 | 0.105 | 0.116 | 0.130 | 0.143 | 0.152 | 0.157 |

* Includes other ancestries (sample N=154) along with the 4 specified ancestral groups.

**Table B**. **SAD/height ratio (SADHtR) for women aged 20 and over and number of examined persons, mean, standard error of the mean, and selected percentiles by ancestry and age: United States, 2011-2014. Estimated population = 105.1 million women.**

|  |  | |  |  |  |  | **Percentile in population** | | | | | | |
| --- | --- | --- | --- | --- | --- | --- | --- | --- | --- | --- | --- | --- | --- |
| **Race and ancestry and age** | | | | **Number of examined persons** | **Population mean** | **Standard error of the mean** | **5th** | **10th** | **25th** | **Median 50th** | **75th** | **90th** | **95th** |
| ***All ancestries**** | | |  |  |  |  |  |  |  |  |  |  |  |
| **20 + years [total]** | | |  | 4945 | 0.1357 | 0.0007 | 0.097 | 0.102 | 0.114 | 0.132 | 0.154 | 0.175 | 0.188 |
| **20-29 years** | | |  | 786 | 0.1241 | 0.0013 | 0.092 | 0.096 | 0.104 | 0.118 | 0.139 | 0.164 | 0.175 |
| **30-39 years** | | |  | 862 | 0.1310 | 0.0011 | 0.095 | 0.099 | 0.109 | 0.126 | 0.149 | 0.171 | 0.181 |
| **40-49 years** | | |  | 922 | 0.1350 | 0.0016 | 0.097 | 0.100 | 0.113 | 0.130 | 0.153 | 0.176 | 0.189 |
| **50-59 years** | | |  | 870 | 0.1419 | 0.0015 | 0.101 | 0.106 | 0.119 | 0.137 | 0.160 | 0.185 | 0.198 |
| **60-69 years** | | |  | 826 | 0.1417 | 0.0009 | 0.105 | 0.111 | 0.122 | 0.138 | 0.158 | 0.179 | 0.189 |
| **70-79 years** | | |  | 442 | 0.1452 | 0.0015 | 0.104 | 0.115 | 0.126 | 0.144 | 0.161 | 0.179 | 0.187 |
| **80 + years** | | |  | 237 | 0.1387 | 0.0016 | 0.107 | 0.111 | 0.124 | 0.138 | 0.154 | 0.167 | 0.177 |
| ***Non-Hispanic white*** | | |  |  |  |  |  |  |  |  |  |  |  |
| **20 + years [total]** | | |  | 1966 | 0.1339 | 0.0008 | 0.097 | 0.101 | 0.112 | 0.130 | 0.152 | 0.173 | 0.187 |
| **20-39 years** | | |  | 620 | 0.1241 | 0.0011 | 0.092 | 0.096 | 0.104 | 0.116 | 0.138 | 0.164 | 0.177 |
| **40-59 years** | | |  | 653 | 0.1362 | 0.0014 | 0.097 | 0.101 | 0.112 | 0.131 | 0.154 | 0.181 | 0.195 |
| **60 + years** | | |  | 693 | 0.1406 | 0.0010 | 0.105 | 0.111 | 0.122 | 0.138 | 0.156 | 0.175 | 0.185 |
| ***Non-Hispanic black*** | | |  |  |  |  |  |  |  |  |  |  |  |
| **20 + years [total]** | | |  | 1137 | 0.1478 | 0.0009 | 0.108 | 0.115 | 0.127 | 0.146 | 0.166 | 0.184 | 0.197 |
| **20-39 years** | | |  | 349 | 0.1421 | 0.0013 | 0.100 | 0.109 | 0.122 | 0.140 | 0.160 | 0.176 | 0.186 |
| **40-59 years** | | |  | 448 | 0.1503 | 0.0015 | 0.111 | 0.117 | 0.128 | 0.148 | 0.168 | 0.188 | 0.200 |
| **60 + years** | | |  | 340 | 0.1531 | 0.0016 | 0.113 | 0.121 | 0.133 | 0.153 | 0.169 | 0.187 | 0.198 |
| ***Hispanic*** | |  |  |  |  |  |  |  |  |  |  |  |  |
| **20 + years [total]** | | |  | 1074 | 0.1400 | 0.0014 | 0.100 | 0.106 | 0.120 | 0.137 | 0.157 | 0.177 | 0.189 |
| **20-39 years** | | |  | 376 | 0.1329 | 0.0019 | 0.098 | 0.102 | 0.114 | 0.130 | 0.149 | 0.170 | 0.178 |
| **40-59 years** | | |  | 396 | 0.1447 | 0.0015 | 0.107 | 0.113 | 0.126 | 0.142 | 0.160 | 0.182 | 0.191 |
| **60 + years** | | |  | 302 | 0.1511 | 0.0017 | 0.115 | 0.122 | 0.132 | 0.150 | 0.167 | 0.186 | 0.195 |
| ***Non-Hispanic Asian*** | | |  |  |  |  |  |  |  |  |  |  |  |
| **20 + years [total]** | | |  | 634 | 0.1206 | 0.0011 | 0.091 | 0.095 | 0.105 | 0.117 | 0.132 | 0.152 | 0.163 |
| **20-39 years** | | |  | 235 | 0.1138 | 0.0013 | 0.090 | 0.092 | 0.099 | 0.110 | 0.125 | 0.137 | 0.150 |
| **40-59 years** | | |  | 248 | 0.1226 | 0.0016 | 0.094 | 0.099 | 0.106 | 0.119 | 0.134 | 0.155 | 0.164 |
| **60 + years** | | |  | 151 | 0.1298 | 0.0018 | 0.099 | 0.106 | 0.115 | 0.127 | 0.139 | 0.159 | 0.171 |

* Includes other ancestries (sample N=134) along with the 4 specified ancestral groups.

**Table C.** **Waist circumference/height ratio (WHtR) for men aged 20 and over and number of examined persons, mean, standard error of the mean, and selected percentiles by ancestry and age: United States, 2011-2014. Estimated population = 103.4 million men.**

|  |  |  | |  |  |  | **Percentile in population** | | | | | | |
| --- | --- | --- | --- | --- | --- | --- | --- | --- | --- | --- | --- | --- | --- |
| **Race and ancestry and age** | | | **Number of examined persons** | | **Population mean** | **Standard error of the mean** | **5th** | **10th** | **25th** | **Median 50th** | **75th** | **90th** | **95th** |
| ***All ancestries**** | |  | |  |  |  |  |  |  |  |  |  |  |
| **20 + years [total]** | |  | | 4949 | 0.5770 | 0.0023 | 0.447 | 0.467 | 0.516 | 0.569 | 0.629 | 0.690 | 0.737 |
| **20-29 years** | |  | | 911 | 0.5307 | 0.0060 | 0.414 | 0.429 | 0.458 | 0.509 | 0.583 | 0.660 | 0.712 |
| **30-39 years** | |  | | 886 | 0.5640 | 0.0033 | 0.449 | 0.467 | 0.506 | 0.547 | 0.608 | 0.675 | 0.720 |
| **40-49 years** | |  | | 835 | 0.5834 | 0.0029 | 0.472 | 0.494 | 0.531 | 0.572 | 0.626 | 0.692 | 0.725 |
| **50-59 years** | |  | | 819 | 0.5919 | 0.0034 | 0.473 | 0.502 | 0.536 | 0.583 | 0.641 | 0.690 | 0.739 |
| **60-69 years** | |  | | 827 | 0.6086 | 0.0059 | 0.491 | 0.504 | 0.548 | 0.600 | 0.658 | 0.720 | 0.775 |
| **70-79 years** | |  | | 440 | 0.6133 | 0.0046 | 0.497 | 0.528 | 0.568 | 0.609 | 0.652 | 0.707 | 0.747 |
| **80 + years** | |  | | 231 | 0.5998 | 0.0041 | 0.503 | 0.523 | 0.558 | 0.596 | 0.638 | 0.683 | 0.706 |
| ***Non-Hispanic white*** | |  | |  |  |  |  |  |  |  |  |  |  |
| **20 + years [total]** | |  | | 1975 | 0.5806 | 0.0026 | 0.451 | 0.472 | 0.520 | 0.572 | 0.633 | 0.694 | 0.736 |
| **20-39 years** | |  | | 700 | 0.5424 | 0.0061 | 0.428 | 0.447 | 0.477 | 0.528 | 0.587 | 0.670 | 0.706 |
| **40-59 years** | |  | | 631 | 0.5911 | 0.0031 | 0.481 | 0.505 | 0.538 | 0.580 | 0.638 | 0.694 | 0.733 |
| **60 + years** | |  | | 644 | 0.6118 | 0.0043 | 0.493 | 0.515 | 0.559 | 0.606 | 0.658 | 0.711 | 0.759 |
| ***Non-Hispanic black*** | |  | |  |  |  |  |  |  |  |  |  |  |
| **20 + years [total]** | |  | | 1145 | 0.5576 | 0.0030 | 0.419 | 0.436 | 0.481 | 0.549 | 0.615 | 0.683 | 0.749 |
| **20-39 years** | |  | | 388 | 0.5276 | 0.0046 | 0.408 | 0.420 | 0.449 | 0.511 | 0.591 | 0.654 | 0.698 |
| **40-59 years** | |  | | 373 | 0.574 | 0.0050 | 0.438 | 0.462 | 0.509 | 0.563 | 0.621 | 0.702 | 0.755 |
| **60 + years** | |  | | 384 | 0.5917 | 0.0054 | 0.460 | 0.483 | 0.537 | 0.585 | 0.638 | 0.701 | 0.757 |
| ***Hispanic*** |  |  | |  |  |  |  |  |  |  |  |  |  |
| **20 + years [total]** | |  | | 1041 | 0.5881 | 0.0042 | 0.460 | 0.489 | 0.536 | 0.583 | 0.635 | 0.691 | 0.738 |
| **20-39 years** | |  | | 376 | 0.5749 | 0.0060 | 0.444 | 0.466 | 0.510 | 0.569 | 0.625 | 0.687 | 0.735 |
| **40-59 years** | |  | | 369 | 0.5981 | 0.0042 | 0.496 | 0.515 | 0.551 | 0.591 | 0.637 | 0.688 | 0.728 |
| **60 + years** | |  | | 296 | 0.6184 | 0.0045 | 0.520 | 0.537 | 0.566 | 0.610 | 0.653 | 0.713 | 0.745 |
| ***Non-Hispanic Asian*** | |  | |  |  |  |  |  |  |  |  |  |  |
| **20 + years [total]** | |  | | 634 | 0.5315 | 0.0032 | 0.435 | 0.451 | 0.484 | 0.530 | 0.574 | 0.615 | 0.641 |
| **20-39 years** | |  | | 249 | 0.5148 | 0.0049 | 0.413 | 0.431 | 0.461 | 0.504 | 0.554 | 0.615 | 0.637 |
| **40-59 years** | |  | | 236 | 0.5371 | 0.0030 | 0.463 | 0.474 | 0.501 | 0.536 | 0.566 | 0.598 | 0.623 |
| **60 + years** | |  | | 149 | 0.5596 | 0.0044 | 0.455 | 0.491 | 0.519 | 0.564 | 0.595 | 0.633 | 0.649 |

* Includes other ancestries (sample N=154) along with the 4 specified ancestral groups.

**Table D**. **Waist circumference/height ratio (WHtR) for women aged 20 and over and number of examined persons, mean, standard error of the mean, and selected percentiles by ancestry and age: United States, 2011-2014. Estimated population = 105.1 million women.**

|  |  |  | | |  |  |  | **Percentile in population** | | | | | | |
| --- | --- | --- | --- | --- | --- | --- | --- | --- | --- | --- | --- | --- | --- | --- |
| **Race and ancestry and age** | | | **Number of examined persons** | | | **Population mean** | **Standard error of the mean** | **5th** | **10th** | **25th** | **Median 50th** | **75th** | **90th** | **95th** |
| ***All ancestries**** | |  | |  | |  |  |  |  |  |  |  |  |  |
| **20 + years [total]** | |  | | 4945 | | 0.5972 | 0.0024 | 0.453 | 0.473 | 0.521 | 0.586 | 0.661 | 0.738 | 0.784 |
| **20-29 years** | |  | | 786 | | 0.5580 | 0.0057 | 0.432 | 0.444 | 0.479 | 0.536 | 0.622 | 0.706 | 0.760 |
| **30-39 years** | |  | | 862 | | 0.5878 | 0.0044 | 0.450 | 0.463 | 0.510 | 0.570 | 0.648 | 0.733 | 0.787 |
| **40-49 years** | |  | | 922 | | 0.5964 | 0.0051 | 0.457 | 0.478 | 0.518 | 0.585 | 0.658 | 0.740 | 0.788 |
| **50-59 years** | |  | | 870 | | 0.6137 | 0.0058 | 0.467 | 0.486 | 0.535 | 0.606 | 0.678 | 0.764 | 0.814 |
| **60-69 years** | |  | | 826 | | 0.6179 | 0.0035 | 0.481 | 0.508 | 0.553 | 0.611 | 0.672 | 0.742 | 0.775 |
| **70-79 years** | |  | | 442 | | 0.6243 | 0.0051 | 0.488 | 0.516 | 0.566 | 0.616 | 0.677 | 0.737 | 0.770 |
| **80 + years** | |  | | 237 | | 0.6012 | 0.0064 | 0.496 | 0.509 | 0.544 | 0.603 | 0.649 | 0.705 | 0.730 |
| ***Non-Hispanic white*** | |  | |  | |  |  |  |  |  |  |  |  |  |
| **20 + years [total]** | |  | | 1966 | | 0.5924 | 0.0028 | 0.453 | 0.470 | 0.516 | 0.580 | 0.654 | 0.732 | 0.779 |
| **20-39 years** | |  | | 620 | | 0.5642 | 0.0046 | 0.441 | 0.454 | 0.482 | 0.537 | 0.619 | 0.712 | 0.771 |
| **40-59 years** | |  | | 653 | | 0.5985 | 0.0046 | 0.457 | 0.477 | 0.518 | 0.583 | 0.661 | 0.751 | 0.798 |
| **60 + years** | |  | | 693 | | 0.6124 | 0.0039 | 0.480 | 0.508 | 0.552 | 0.608 | 0.665 | 0.728 | 0.756 |
| ***Non-Hispanic black*** | |  | |  | |  |  |  |  |  |  |  |  |  |
| **20 + years [total]** | |  | | 1137 | | 0.6268 | 0.0031 | 0.466 | 0.498 | 0.548 | 0.619 | 0.691 | 0.769 | 0.808 |
| **20-39 years** | |  | | 349 | | 0.6113 | 0.0052 | 0.434 | 0.473 | 0.534 | 0.601 | 0.677 | 0.767 | 0.805 |
| **40-59 years** | |  | | 448 | | 0.6341 | 0.0054 | 0.478 | 0.504 | 0.550 | 0.622 | 0.699 | 0.775 | 0.811 |
| **60 + years** | |  | | 340 | | 0.6409 | 0.0060 | 0.497 | 0.524 | 0.574 | 0.636 | 0.702 | 0.757 | 0.801 |
| ***Hispanic*** |  |  | |  | |  |  |  |  |  |  |  |  |  |
| **20 + years [total]** | |  | | 1074 | | 0.6162 | 0.0049 | 0.471 | 0.498 | 0.544 | 0.611 | 0.673 | 0.745 | 0.786 |
| **20-39 years** | |  | | 376 | | 0.5954 | 0.0070 | 0.454 | 0.479 | 0.524 | 0.589 | 0.657 | 0.728 | 0.764 |
| **40-59 years** | |  | | 396 | | 0.6292 | 0.0051 | 0.497 | 0.516 | 0.565 | 0.622 | 0.680 | 0.752 | 0.800 |
| **60 + years** | |  | | 302 | | 0.6519 | 0.0055 | 0.526 | 0.547 | 0.598 | 0.643 | 0.704 | 0.762 | 0.800 |
| ***Non-Hispanic Asian*** | |  | |  | |  |  |  |  |  |  |  |  |  |
| **20 + years [total]** | |  | | 634 | | 0.5436 | 0.0036 | 0.429 | 0.450 | 0.488 | 0.536 | 0.589 | 0.651 | 0.692 |
| **20-39 years** | |  | | 235 | | 0.5166 | 0.0054 | 0.420 | 0.429 | 0.462 | 0.504 | 0.557 | 0.609 | 0.660 |
| **40-59 years** | |  | | 248 | | 0.5545 | 0.0041 | 0.449 | 0.470 | 0.506 | 0.538 | 0.598 | 0.663 | 0.698 |
| **60 + years** | |  | | 151 | | 0.5741 | 0.0058 | 0.468 | 0.489 | 0.530 | 0.565 | 0.609 | 0.674 | 0.707 |

* Includes other ancestries (sample N=134) along with the 4 specified ancestral groups
