# Supplementary figures and images for: Indicators of abdominal size relative to height associated with sex, age, socioeconomic position and ancestry among US adults
Source: PLoS One. 2017 Mar 1;12(3):e0172245. doi: 10.1371/journal.pone.0172245 (PMC5332027; doi:10.1371/journal.pone.0172245)

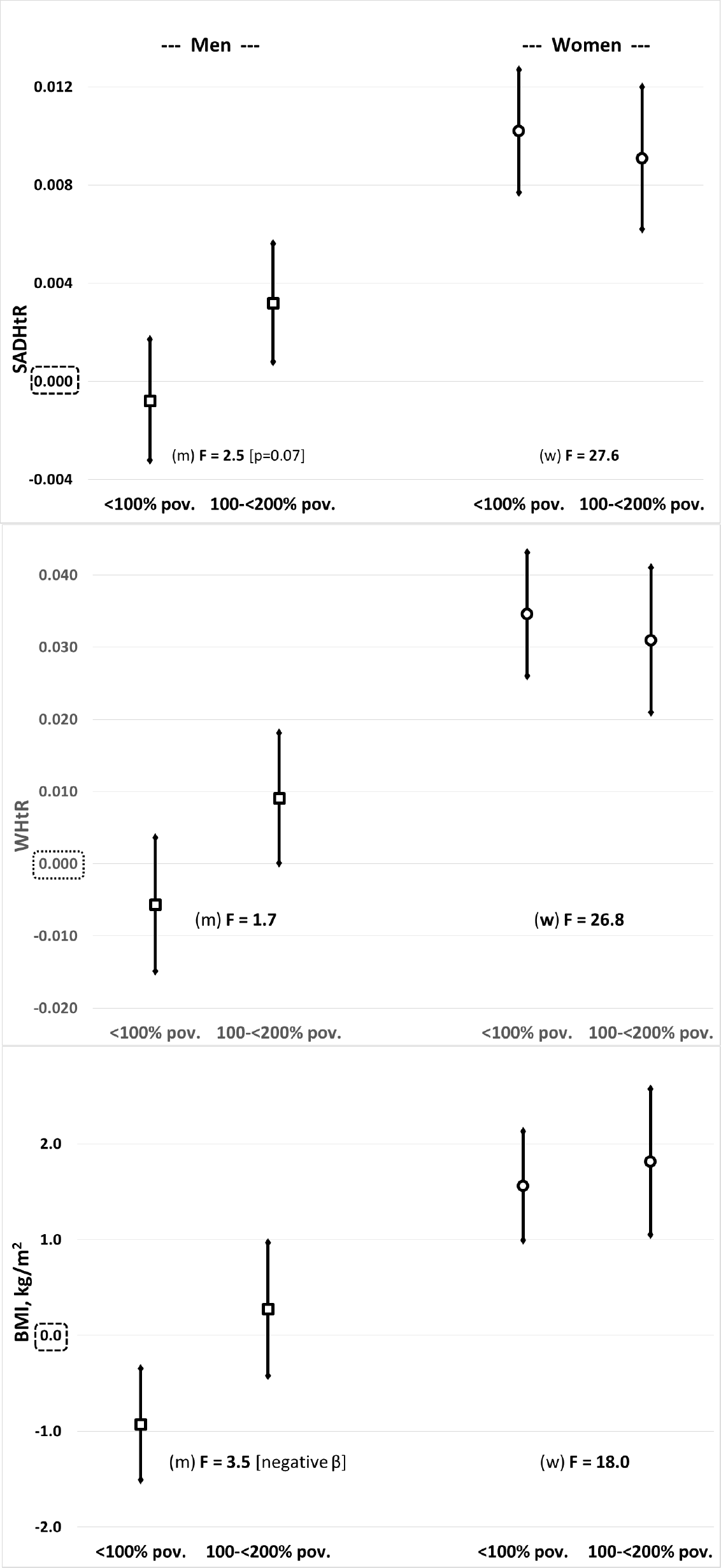

Supplement: S1 Fig — Separate models, each adjusted for age and ancestral groups, were prepared for [top] SADHtR, [middle] WHtR, and [bottom] BMI. F indicates the adjusted Wald F statistic, a summary effect describing confidence that contrasts in family income (3-levels) have influenced the observed value for the adiposity indicator. These models include a category of persons (7%) with unknown family income. (TIF) [file pone.0172245.s002.tif]
